# CFD Analysis of the Flow Field in an SBS Furnace Under Thick Slag Layer Conditions for Nickel Smelting

**DOI:** 10.3390/ma19132882

**Published:** 2026-07-06

**Authors:** Kezhou Song, Pekka Taskinen, Yuanmin Zou, Zhigang Liu, Yingde Huang, Can Ji, Cong Wang, Shenglong Zhang, Fuqiang Xu

**Affiliations:** 1 Energy Research Institute, Qilu University of Technology (Shandong Academy of Sciences), Jinan 250014, China; kezhou.song@qlu.edu.cn (K.S.);; 2 School of Chemical Engineering, Aalto University, 02150 Espoo, Finland; pekka.taskinen@aalto.fi; 3 School of Materials Science and Engineering, Zhengzhou University, Zhengzhou 450001, China

**Keywords:** Multi-Fluid VOF, laterite, side blowing, Vanyukov furnace, numerical simulation

## Abstract

The demand for metallic nickel has been growing steadily. Laterite nickel ore, as an oxide mineral, contains a higher nickel content than sulfide ores, enabling more economical smelting in the nickel extraction process. However, an extremely thick slag layer generated during side-blown smelting of laterite nickel ore alters the bath circulation characteristic of conventional smelting processes. To address this, this study employs a validated Multi-Fluid VOF model to analyze the flow field characteristics of the side-blown furnace bath under varying slag layer thicknesses. Within the slag thickness range of 1.5–2.5 m, the ascending air plume shifts horizontally along the furnace wall with increasing slag thickness, entraining matte into the slag and promoting its diffusion toward the settling zone. Matte diffusion is governed by bath circulation: backflow from the settling zone dominates at lower slag thickness, while vertical kinetic energy from splashed melt reflux dominates at higher thickness. This reflux-induced circulation is blocked by the dense matte layer, causing no disturbance to the bottom matte and thus favoring slag–matte separation. Increasing slag thickness raises the near-wall circulation zone and plume entry point, reducing gas holdup near the tuyere and weakening oxygen–matte contact.

## 1. Introduction

The nickel resources in lateritic (oxide) deposits are much larger than in sulphidic minerals [1] and their use along with growing nickel demand is increasing. Smelting nickel laterite to matte is an existing technology [2] but due to the high iron-to-nickel ratio and high silica and magnesia concentrations, the smelting is energy intensive and requires high temperatures if reduced to an iron-free slag end-point [3]. The Vanyukov furnace technology has been tested for oxidic nickel ores in the past [4,5,6] and its option for direct sulfidation would benefit the process with a higher nickel concentration in the product matte compared with sulfidation using sulfide minerals [7,8]. An essential feature of most nickel laterite ores is a high moisture and crystalline water content [9] which must be removed prior to smelting with energy efficient technologies, e.g., by steam drying and rotary kiln technologies, for lowering energy usage.

The smelting process of lateritic nickel ore can be realized by side-blown technology. For furnaces with double-side blowing referred to as side-blown smelting furnaces (SBS furnace), such as the Vanyukov furnace, two rows of tuyeres are positioned on opposite sides of the furnace. The agitation zone generated by the injected oxygen-enriched air differs from that of submerged top blowing or bottom blowing processes, being primarily distributed near the wall regions. Since the velocity of the injected gas is insufficient to form a jet that penetrates the high-density melt, the bubbling does not cause strong agitation in the central melt region. Instead, mass diffusion within the melt is promoted through processes such as melt bath circulation [10]. Air circulation accelerates the formation of nickel matte and slag during the laterite nickel ore smelting process, but at the same time causes further mixing of the matte and slag, potentially increasing the chemical and mechanical entrainment of nickel matte in the slag. Compared with high-speed jet systems such as oxygen-enriched bottom blowing, the flow field in the side blowing process is relatively moderate, contributing to a longer service life and thereby facilitating its widespread application [11]. Nevertheless, the transport processes within the melt bath are still not fully understood [10]. SBS furnaces are predominantly used in copper smelting processes, and current research has therefore focused primarily on flow field analyses of copper smelting furnaces [10].

The gas flow pattern in SBS furnaces is primarily characterized by air plumes [12]. Prediction of the internal flow field structure generally relies on computational fluid dynamics (CFD) methods based on the Volume of Fluid (VOF) model, as well as scaled-down water model observations [10]. As a conventional multiphase flow model, the VOF model has been widely employed to predict melt bath flow in side-blown furnaces, including Vanyukov furnaces and Peirce–Smith converters [13,14,15]. Wu et al. validated VOF simulation results using a water model and further applied the model to simulate the melt bath flow field and calculate gas holdup [16]. Xiao et al. used a water model combined with a high-speed camera to track bubble motion, observing small-scale circulation near the melt surface induced by the plume [17]. Based on water model experiments and VOF predictions, Wan et al. found that the melt bath flow in side-blown furnaces is closely related to the gas flow rate [18]. In addition, Bian et al. enhanced interface tracking using a Coupled Level Set–VOF model, which improved simulation accuracy and elucidated the velocity field in the melt bath under various tuyere configurations [19]. The tuyere structure was also investigated by Xiao et al., focusing on bubble behavior with swirl tuyeres [20]. Systematic bubble characteristics were revealed by Li et al. [21]. Furthermore, based on CFD simulation results, a rapid prediction model has been established to predict the bath flow field [22].

Current research on transport processes in SBS furnaces has generally focused on the relatively typical copper metallurgy process, rendering it not entirely applicable to the smelting of laterite nickel ore, which involves a substantial slag layer. In view of state of research, this simulation targets the presence of an extremely thick slag layer during the laterite nickel ore smelting process, which differs significantly from the copper concentrate smelting process. Given the requirement for interfacial force model accuracy in predicting side-blown plume motion, this study adopts the Multi-Fluid VOF multiphase flow model, which has demonstrated good predictive accuracy for gas jet penetration depth and motion morphology under both bottom-blown and side-blown conditions [23,24,25,26]. This study will reveal the movement behavior of the plume, the slag–matte mixing performance, the characteristics of macroscopic circulation in the bath, and the changes in gas holdup induced by circulation under the condition of an extremely thick slag layer, thereby improving the understanding of the nickel ore smelting process in the SBS furnace.

## 2. Mathematical Model

The numerical simulation scheme for side-blown furnaces mainly considers strong plume flow, which is significantly different from scenarios such as slow bubble flow and small-scale seepage flow [27,28,29]. The gas–matte–slag multiphase system examined in this CFD simulation closely replicates the fundamental characteristics of the molten bath in an oxygen-enriched bottom-blown copper furnace. Both systems are driven by an injected gas plume that induces melt circulation, with analogous physical properties and interphase dynamics. Accordingly, this study adapts the established multiphase numerical framework [25] developed for bottom-blown smelting furnaces, using Multi-Fluid VOF model for the multiphase flows with particular emphasis on inertial effects under high gas injection rates. Key interfacial forces governing the flow are incorporated to enable accurate prediction of bubble plume behavior and the trajectories of sulfide matte and slag phases.

### 2.1. Governing Equations

The mass and momentum conservation equations for phase q (including the gas phase and liquid phase) are given as Equations (1) and (2), respectively:

(1) $$\frac{\partial }{\partial t}\left(\alpha_{q}\rho_{q}\right)+\nabla \cdot \left(\alpha_{q}\rho_{q}{\vec{v}}_{q}\right)=0$$

(2) $$\frac{\partial }{\partial t}\left(\alpha_{q}\rho_{q}{\vec{v}}_{q}\right)+\nabla \cdot \left(\alpha_{q}\rho_{q}{\vec{v}}_{q}{\vec{v}}_{q}\right)=-\nabla p+\nabla \cdot \left[\mu \left(\nabla {\vec{v}}_{q}+{\nabla {\vec{v}}_{q}}^{T}\right)\right]+\alpha_{q}\rho_{q}\vec{g}+\vec{F}$$

(3) $$\alpha_{q}=\alpha_{g}\;or\;\alpha_{l1}\;or\;\alpha_{l2},\alpha_{g}+\alpha_{l1}+\alpha_{l2}=1$$

In Equations (1)–(3), α is the volume fraction, ρ is the density,$\vec{v}$ is the velocity, p is the pressure shared by both two phases, $\alpha_{q}\rho_{q}\vec{g}$ is the gravity term, and $\vec{F}$ is an external body force which is specifically defined as the drag force in the current system. The subscripts *g* and *l* represent the gas and liquid phases, respectively.

### 2.2. Interface Forces

Given the high relative velocities and significant interfacial slip at the gas–liquid interface in the side-blown furnace under investigation, the interfacial drag force and virtual mass force are prioritized for their substantial effects on multiphase flow dynamics. Other interfacial forces, which do not significantly influence macroscopic flow behavior, are neglected in this simulation to enhance computational efficiency. This treatment of interfacial force modeling has been previously validated through water modeling experiments [25].

Drag force is implemented utilizing the symmetric model provided by the Multi-Fluid VOF model [30]. For the symmetric model, density and viscosity are calculated from volume averaged properties:

(4) $$\rho_{gl}=\alpha_{g}\rho_{g}+\alpha_{l}\rho_{l}$$

(5) $$\mu_{gl}=\alpha_{g}\mu_{g}+\alpha_{l}\mu_{l}$$

and the diameter of bubbles or droplets is defined as

(6) $$d=\frac{1}{2}\left(d_{q1}+d_{q2}\right),\;d_{q}=d_{g}\;or\;d_{l}\;$$

In turn, the drag function can be given as

(7) $$f=\frac{C_{D}Re}{24}$$

where the relative Reynold number *Re* is

(8) $$Re=\frac{\rho_{gl}\left|{\vec{v}}_{g}-{\vec{v}}_{l}\right|d_{gl}}{\mu_{gl}}$$

and the drag coefficient *C_D_* is

(9) $$C_{D}=\left\{\begin{array}{l} 24\left(1+0.15{Re}^{0.687}\right)/Re\;\;\;\;\;\;\;\;Re\leq 1000\\ 0.44\;\;\;\;\;\;\;\;\;\;\;\;\;\;\;\;\;\;\;\;\;\;\;\;\;\;\;\;\;\;\;\;\;\;\;\;\;\;\;\;\;\;\;\;Re\geq 1000 \end{array}\right.$$

In highly dynamic flow fields, the virtual mass force effectively captures the additional inertial forces due to fluid acceleration. This consideration becomes particularly relevant in contexts where materials of different densities undergo accelerated relative motion.

(10) $${\vec{F}}_{vm}=0.5\alpha_{q}\rho_{q}\left(\frac{d_{q}{\vec{v}}_{q}}{dt}-\frac{d_{p}{\vec{v}}_{p}}{dt}\right)$$

The term $\frac{d_{q}}{dt}$ denotes the phase material time derivative of the form:

(11) $$\frac{d_{q}\left(\emptyset \right)}{dt}=\frac{\partial \left(\emptyset \right)}{\partial t}+\;\left({\vec{v}}_{q}\cdot \nabla \right)\emptyset$$

### 2.3. Turbulence Models

The standard k-ε turbulence model with standard wall functions was adopted for the present simulation. The kinetic energy k and its rate of dissipation ε yields are as follows:

(12) $$\frac{\partial }{\partial t}\left(\rho_{m}k\right)+\nabla \cdot \left(\rho_{m}{\vec{v}}_{m}k\right)=\nabla \cdot \left(\alpha_{l}\frac{\mu_{t}}{\sigma_{k}}\nabla k\right)+G_{k}+G_{b}-\rho_{m}\varepsilon ,$$

(13) $$\frac{\partial }{\partial t}\left(\rho_{m}\varepsilon \right)+\nabla \cdot \left(\rho_{m}{\vec{v}}_{m}\varepsilon \right)=\nabla \cdot \left(\frac{\mu_{t}}{\sigma_{k}}\nabla \varepsilon \right)+\frac{\varepsilon }{k}\left(C_{1\varepsilon }\left(G_{k}+G_{b}\right)\right)-\frac{\varepsilon^{2}}{k}C_{2\varepsilon }\rho_{m,}$$

where G_k_ represents the generation of turbulence energy due to the mean velocity gradients, and G_b_ is the turbulence energy due to buoyancy. The mixture density $\rho_{m}\;$ and mixture velocity ${\vec{v}}_{m}$ are defined as follows:

(14) $$\rho_{m}=\alpha_{g}\rho_{g}+\alpha_{l1}\rho_{l1}+\alpha_{l2}\rho_{l2}$$

(15) $${\vec{v}}_{m}=\frac{\alpha_{g}\rho_{g}{\vec{v}}_{g}+\alpha_{l1}\rho_{l1}{\vec{v}}_{l1}+\alpha_{l2}\rho_{l2}{\vec{v}}_{l2}}{\alpha_{g}\rho_{g}+\alpha_{l1}\rho_{l1}+\alpha_{l2}\rho_{l2}}$$

The empirical constants are $C_{1\varepsilon }=1.44$, $C_{2\varepsilon }=1.92$, $\sigma_{\varepsilon }=1$, $\sigma_{k}=1$. The turbulence viscosity $\mu_{t}$ is given as Equation (14):

(16) $$\mu_{t}=\rho_{m}C_{\mu }\frac{k^{2}}{\varepsilon },C_{\mu }=0.09$$

## 3. Geometry and Simulation Conditions

### 3.1. Physical Model

**Figure 1:**
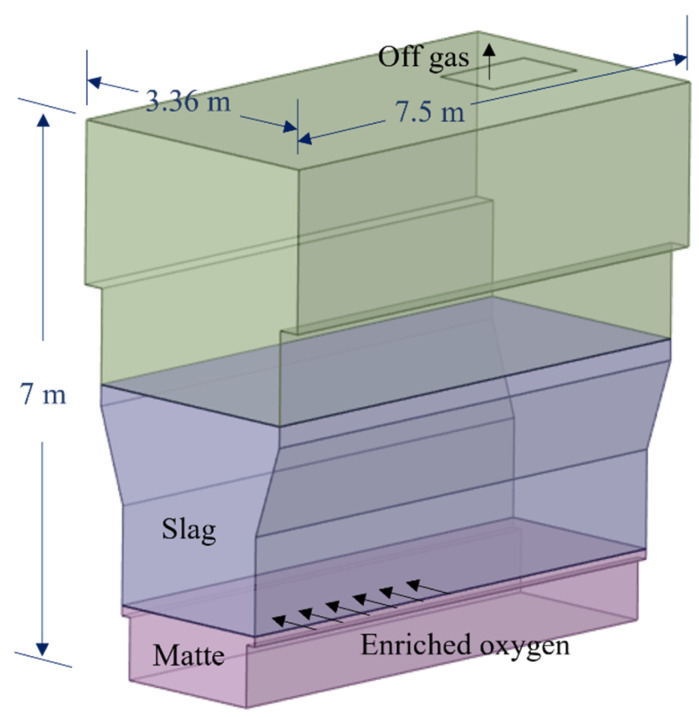
Physical model of the simulated SBS furnace.

The structure of the SBS furnace used for laterite nickel ore smelting to matte does not differ significantly from common side-blown furnace types, such as the Vanyukov furnace. Its distinct characteristic lies in the significantly greater slag layer thickness during nickel laterite smelting compared to copper smelting processes. Therefore, the parameters of the full-scale physical model in this study are based on a typical SBS furnace [25], with six oxygen tuyeres distributed at a low level of the furnace, to promote the agitation of the slag and accelerate the reactions. While maintaining a constant matte layer thickness, slag layers of varying thicknesses ranging from 0.5 m to 1.5 m are set as variables to investigate the influence mechanisms of thick slag layers on phase distribution and velocity fields. A physical model for the simulated furnace is shown in Figure 1, presenting a common situation with a thick slag layer in the bath for nickel smelting processes. The operating parameters for the simulated furnace are presented in Table 1. During the nickel smelting process, the nickel concentrate is continuously fed onto the agitated surface of the slag, where a series of chemical reactions take place, producing the matte of metallic sulfides. Due to its higher density, the matte moves to the bottom of the furnace under the effect of gravity, from where it is continuously removed. The kinetics of these reactions and the efficiency of matte separation depend on the fluid dynamics present in both the slag and the matte.

To improve calculation efficiency, the following simplifications were implemented in the physical model:

(1) The furnace walls are simplified as smooth surfaces, neglecting the minor influences of intricate geometric details on the macro-scale flow.
(2) Given the substantial air injection volume and high air injecting velocity in the SBS furnace, priority is given to the macro-scale motion dominated by inertial effects. Variations in physical properties induced by temperature fluctuations and phase transformations only introduce negligible perturbations at interfaces, which do not affect the conclusions of this study and were therefore disregarded.

### 3.2. CFD Model

**Figure 2:**
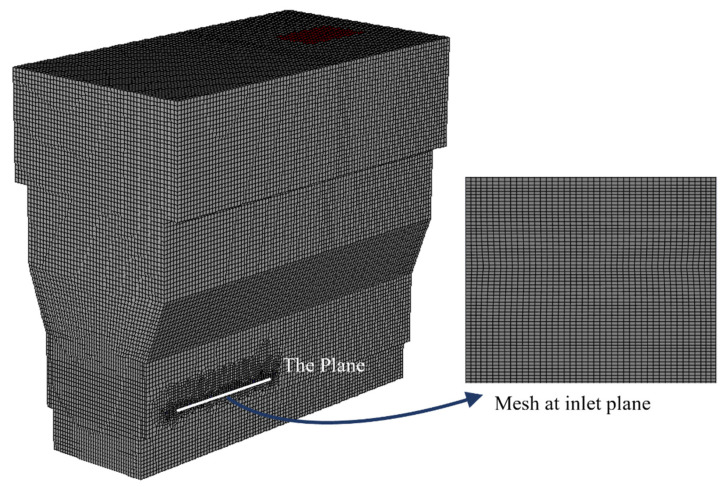
Mesh of the simulated SBS furnace.

The CFD configuration employed in this study aligns with prior investigations, selected for its computational efficiency and reliability [25,26]. Figure 2 illustrates the mesh topology applied to the simulated SBS furnace. A hexahedral grid was constructed via multizone meshing to enhance quality along the vertical direction. Since this research does not focus on small-scale phenomena such as detailed bubble dynamics, a mesh simplification strategy was employed relative to conventional approaches, enabling accurate prediction of macroscopic flow characteristics over extended durations. To improve both mesh quality and computational performance, the original gas inlets, which are notably small (38 mm in diameter, approximately 1/200 of the furnace length), were geometrically simplified and squared, consistent with earlier treatments. This simplification was validated through a grid independence test, confirming that the macroscopic velocity profile and wall shear stress remain insensitive to both the inlet treatment and mesh resolution [26]. The final mesh consists of approximately 500,000 cells, with over 99.68% of elements exhibiting skewness below 0.50, indicating good to excellent quality, while the remaining 0.32% show skewness under 0.54, rated as acceptable [32]. The locally refined inlet mesh demonstrates satisfactory convergence behavior and has been verified against experimental data for accuracy [25,26].

As presented in Figure 1 and Figure 2, the gas inlets are located at the furnace bottom (within a refined grid region) and defined as velocity inlets. The outlet is positioned at the top surface, away from the agitation zone, and set as a pressure outlet. A time step size of 5 × 10^−4^ s was adopted, with convergence criteria set at dimensionless residuals below 1 × 10^−3^.

### 3.3. Model Validation

For submerged injection furnace types, including SBS furnaces and bottom-blown (SKS) furnaces, a substantial volumetric flow rate of air is introduced through oxygen tuyeres with diameters of several tens of millimeters. This results in a high initial air velocity entering the molten bath, forming a gas plume under the resistance of high-density, high-viscosity melt. To characterize this flow behavior, the authors previously developed a numerical framework for horizontal cylindrical bottom-blown and side-blown furnaces, combining a Multi-Fluid VOF model with interfacial resistance and virtual mass force. This approach successfully predicted the vertical and horizontal velocity vector components of the gas plume in the multiphase flow system of submerged injection furnaces and accurately captured the periodic oscillations in the bath flow field. In the present study, the SBS side-blown furnace features a polygonal structure, with gas injected vertically to the wall, like the large-angle tuyere configuration in P-S converters or SKS furnaces. The most critical validation basis for the vertical-wall blowing mode is the penetration depth, i.e., the horizontal component of the plume displacement when the cylindrical furnace is blown at an angle more than 0°. In previous cases of more complex cylindrical furnace blowing, it has been demonstrated on multiple occasions that the horizontal displacement of the plume is consistent with the physical model. Therefore, the previously validated multiphase flow model for the bottom and side-blown furnace has been adopted in this work, which has been thoroughly verified [25,26,27].

## 4. Results and Discussion

**Figure 3:**
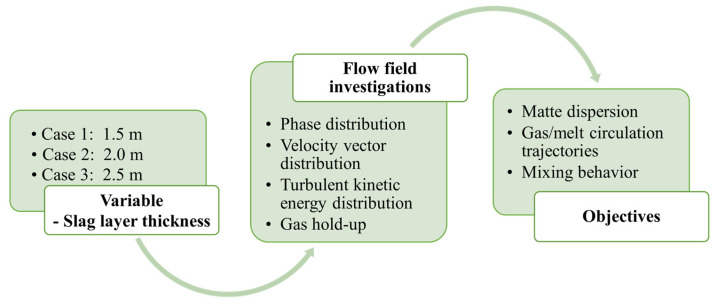
A guideline for this study including the variable, investigations and objectives.

In the smelting process of laterite nickel ore, an extremely thick slag layer exists, which becomes the primary liquid phase in the bath and prolongs the upward path of incident gas plumes. To reveal the mechanism in terms of flow behavior with an existence of different slag layer thicknesses, flow fields in the simulated furnace at conditions of 1.5 m, 2.0 m, and 2.5 m slag layers are investigated. A guideline for this study is shown in Figure 3.

### 4.1. Plume Motion Characteristics

**Figure 4:**
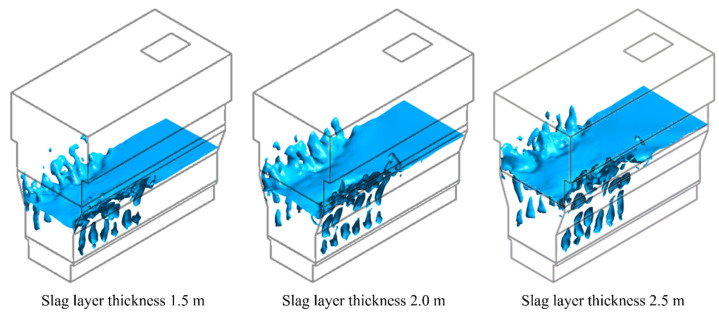
Gas–liquid interface in simulated furnaces at conditions of different slag layer thicknesses.

In the Vanyukov and SBS furnaces, the sidewall near the oxygen tuyeres is not vertical but features multiple trapezoidal inclined surfaces extending outward, potentially influencing the trajectory of ascending air. Therefore, to observe the movement trajectory of the plume, this study first extracts the gas–liquid interface at a gas volume fraction of 0.5 from the simulation results, as shown in Figure 4. According to Figure 4, as the slag layer thickness increases, the trapezoidal inclined surfaces covered by the slag layer gradually expand, while the air plumes under different slag layer thicknesses consistently ascend along the wall, instead of climbing vertically, exhibiting significant wall-attachment effect characteristics. In the thick slag layer system, the bath width gradually expands based on the trapezoidal structure of the furnace, thereby reducing the contact between the central region and the gas plumes on both sides. Consequently, the melt in the centerline area gets less mixing efficiency with the injected oxygen, and the wall is under an impact from a mix of both slag and air phase. In matte making from laterites, the reactions are endothermic and thus fuel must be combusted in the slag phase to maintain temperature, the metal generating reactions, and sulfide matte formation in the vessel.

**Figure 5:**
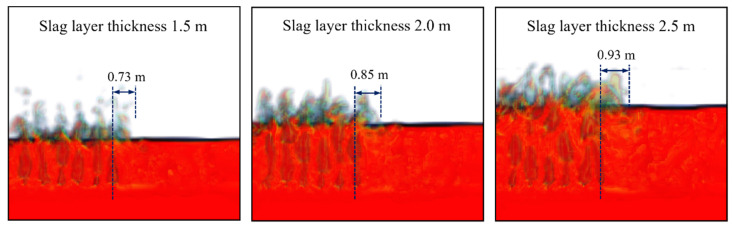
Air plumes and splashing in simulated furnaces at conditions of different slag layer thicknesses.

For clear observation of the motion patterns of the gas plume, Figure 5 presents a side view of the oxygen tuyere side of the molten pool density distribution. In the case of a 1.5 m slag layer thickness, the gas plume maintains relatively good continuity during its ascent. However, under the 2.0 m condition, the continuity of the plume is disrupted by the wall-attachment effect along the inclined inner wall, leading to segmentation of the plume. Under the 2.5 m condition, the plume experiences more pronounced disturbance, resulting in widespread fragmentation and displacement toward both ends of the molten pool. This plume fragmentation and inclination cause differences in splashing directions. For a 1.5 m thick slag layer, the splashed melt is ejected nearly vertically upward. In contrast, under the 2.5 m slag layer condition, influenced by the inclined plume, significantly more lateral splashing occurs, causing the lateral distance to increase from 0.73 m to 0.93 m. These differing splashing directions may lead to variations in the circulation patterns within the molten pool.

### 4.2. Slag/Matte Mixing and Bath Circulation

**Figure 6:**
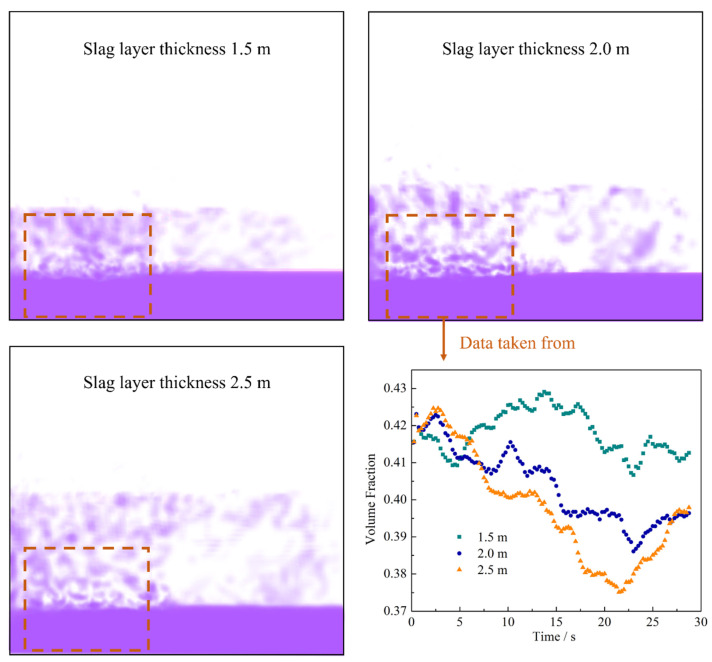
Nickel matte distributions in simulated furnaces at conditions of different slag layer thicknesses. The chart presents the matte volume fraction in the marked area.

Variations in slag layer thickness leads to alterations in the trajectories of both the plume and splashed melts, which consequently changes the movement paths of the matte phase entrained within the slag layer. Figure 6 illustrates the distribution of nickel matte, demonstrating how nickel matte droplets, dragged by ascending bubbles and flowing slag, disperse within the slag layer. As the slag layer thickness increases, the distribution of nickel matte becomes progressively more dispersed. This could be more clearly presented by the chart in Figure 6, which shows that the matte volume fraction in the marked tuyere nearing region decreased with the improving of slag layer thickness. Moreover, with a thicker slag layer, the matte droplets consistently reach the farther region of the reaction zone and exhibit a tendency to gradually approach the wall close to the tapping end.

This trend may enhance the nickel content within the slag and is likely associated with the macroscopic flow circulation of the bath under different slag thicknesses. In Figure 6, the lateral displacement of the plume strengthens continuously with increasing slag layer thickness. This enhanced displacement promotes a significant intensification of bath circulation under thicker slag conditions. Consequently, in addition to being carried upward by the plume, the nickel matte undergoes further lateral movement, migrating towards the far side region of the reaction zone.

**Figure 7:**
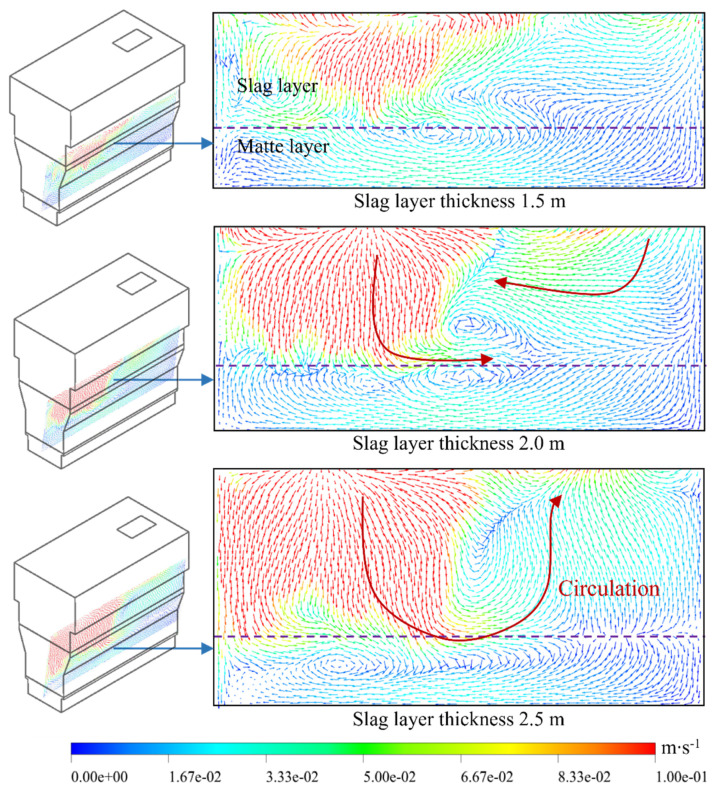
Matte velocity vectors in simulated furnaces at conditions of different slag layer thicknesses. Small arrows point to the movement direction of nickel matte, and its position is located at the middle cross-section of the furnace body. Large arrows indicate how the circulation formed.

The changes in the behavior of the matte phase within the slag layer as its thickness increases are explained by the velocity vector distribution in Figure 7. As shown in Figure 7, for slag layer thicknesses ranging from 2.0 to 2.5 m, the impact caused by the recirculation of splashed melts in the main reaction zone gradually intensifies and expands with increasing slag thickness. This indicates more vigorous melts movement within the reaction zone under thicker slag conditions, explaining why the distribution of nickel matte in the slag layer is more dispersed compared to the 1.5 m case. Under thicker slag conditions, the settling zone (marked in Figure 7) is also more active than in the 1.5 m case. The difference between slag layer thicknesses of 2.0 m and 2.5 m lies in the dominant flow mechanisms. For the 2.0 m slag layer, backflow in the settling zone plays the leading role, which is represented in the figure as the primary cause for the formation of the regional circulation. In contrast, for the 2.5 m case, a large-scale circulatory flow develops within the bath. The flow direction of the melts in the settling zone is opposite to that observed in the 2.0 m case.

According to Figure 5, at a slag thickness of 2.5 m, the lateral displacement of the splashed melts is most prominent. The material that splashes away from the reaction zone and subsequently falls back is highly likely to cause downward movement of the melts in the settling zone. This suggests the flow pattern could potentially evolve into a situation similar to the 2.0 m case. The fact that the flow direction is completely opposite to that in the 2.0 m case indicates that, for the 2.5 m slag layer, the vertically falling-back material into the reaction zone is the primary driver of the large-scale bath circulation. The splashed material that undergoes lateral displacement away from the reaction zone is impeded by this dominant large-scale circulation upon its return to the bath, preventing the formation of a widespread, large-scale downward macroscopic flow.

### 4.3. Flow Field Structure and Bath Gas Holdup

**Figure 8:**
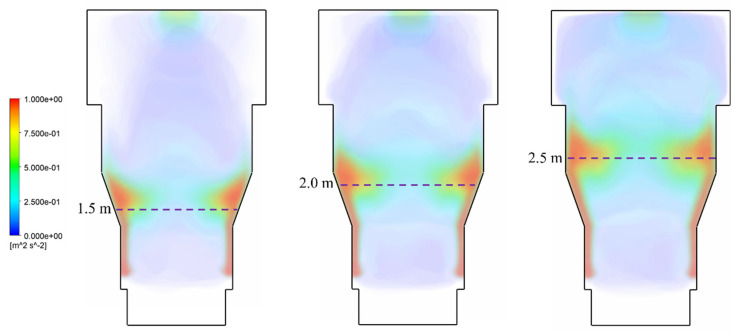
Turbulent intensity in simulated furnaces at conditions of different slag layer thicknesses. The maximum turbulent intensity for each condition happened at the slag level nearing the wall.

The bubble plume serves as the primary kinetic energy source driving molten bath circulation, and its trajectory significantly influences the macroscopic flow pattern within the bath. The distribution of turbulent kinetic energy (TKE) is shown in Figure 8, from a side-view perspective at different slag layer depths. Each image represents a composite overlay of 21 TKE distribution images at 95% transparency, captured at 0.5 s intervals from 20 to 30 s of the simulated furnace operation.

As shown in Figure 8, the TKE distributions under different slag layer thicknesses exhibit remarkably similar characteristics. The TKE in the bottom matte layer approaches zero, indicating minimal disturbance in this slag–matte interface region across all three conditions. The impact of the plume is thus predominantly concentrated within the slag layer, especially near the slag–atmosphere interface. Despite the varying lengths of submerged vertical and inclined walls across the three conditions, the plume trajectory is not significantly altered by the wall structure due to a pronounced wall-adherence effect inducing by fluid viscosity. The plume ascends along the wall, resulting in the highest turbulence intensity in the near-wall region.

The distinction among different slag depths lies in the enhanced turbulence intensity of the melt near the bath surface as the slag layer thickness grows. This observation aligns with the increased horizontal displacement of the splashed melts noted in Figure 5.

**Figure 9:**
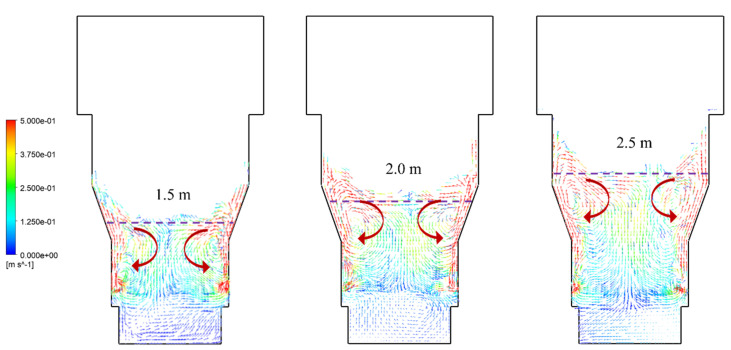
Side view of velocity vectors in simulated furnaces at conditions of different slag layer thicknesses. Large arrows indicate the circulation zones generated by air plumes on both sides of the bath.

To elucidate the formation mechanism of the turbulent kinetic energy (TKE) distribution, Figure 9 presents the side-view vector distribution of melt flow within the furnace. Analysis of Figure 9 reveals that the incident bubble plumes in all three cases generate an essentially similar bath circulation pattern. Specifically, the plume ascends along the wall, drags melt upward away from the bath surface, and then causes splashing toward the central region of the bath. The material returning to the surface forms a small-scale recirculation zone, which subsequently drives a downward flow of melt in the center line region of the furnace. This is in accordance with the observation from Wang et al. [33].

The downward-moving flow, predominantly composed of slag, undergoes significant flow splitting upon encountering the underlying high-density matte layer. It is evident that the reduced downward velocity is insufficient to create a substantial impact on the bottom matte layer. The melt diverted horizontally is then entrained by the incident bubble plume, ascends again, and re-enters the circulation loop. This circulatory motion helps enhance reaction efficiency by promoting more thorough chemical reactions within the bath.

Minor differences exist in both the horizontal and vertical displacements of the melt under different slag thicknesses, leading to variations in gas holdup. In this simulation, a region near the tuyere within the reaction zone was selected as the sampling area for gas holdup variation curves. This area is located below the bath surface and close to the bottom nickel matte layer (or high-density phase), representing the primary zone for bath chemical reactions. The same location was sampled for all three cases with different depths, thereby eliminating other interfering factors. Under these conditions, gas holdup variations arise solely from the influence of internal bath flow.

**Figure 10:**
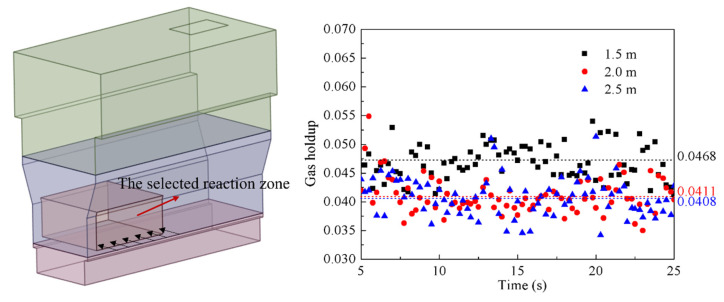
Gas holdup of a selected reaction zone in simulated furnaces at conditions of different slag layer thicknesses. The reaction zone is selected near the tuyeres so that it is contained within all three simulated baths, which have different slag layer thicknesses.

The gas holdup variations in the tuyere nearing area for the three bath depths are shown in Figure 10. The gas holdup levels for slag depths of 2.0 m and 2.5 m are similar and significantly lower than that for the shallower 1.5 m slag layer. In all three cases, the bubble plume ascends along the wall, exerting minimal direct influence on the central part of the reaction zone. Therefore, the analysis of gas holdup must be combined with the melt flow vector diagrams. According to Figure 9, the two recirculation zones in the 1.5 m case are positioned lowest and partially extend into the gas holdup measurement area. In contrast, for the 2.0 m and 2.5 m cases, the recirculation zones are situated farther from the bottom. Air bubbles entering these zones cannot descend further, resulting in lower gas holdup compared to the 1.5 m case, with similar levels between the two thicker slag cases. Overall, the differences in gas holdup near the tuyere level are mainly attributed to the varying positions of the circulation region under different slag layer conditions. For the investigated cases, areas of the gas circulation regions are roughly similar. When the slag layer is thicker, the longer rising path may result in a slightly longer residence time of the oxygen-enriched air in the melt. However, since the circulation region is farther from the matte layer, this may lead to a decreased efficiency to the sulfur removal.

Based on Figure 6, the matte phase (or a high-density phase) is better dispersed under thicker slag conditions but relatively concentrated in the 1.5 m case. Considering the gas holdup distribution characteristics, a thinner slag layer appears more conducive to achieving efficient mixing between the high-density phase and air within a confined space.

The above analysis provides a reaction engineering reference for industrial practice concerning matte–slag separation and sulfur removal efficiency. Specifically, under conditions of a thick slag layer, the practical issue of increased nickel content in the slag may arise. In future designs, the effect of a separating wall should be considered to hinder the diffusion of nickel matte toward the settling zone and to enhance melt circulation in the reaction zone.

## 5. Conclusions

A numerical simulation is conducted to predict the bath flow field in a side-blown nickel smelting furnace featuring an extremely thick slag layer, which helps us to understand the matte loss and sulfur removal in nickel smelting practice. The findings are as follows:

(1) Within the slag thickness range of 1.5–2.5 m, the air plume ascends along the vessel wall in all cases, exhibiting a progressively increasing trend of horizontal displacement. Entrained by the plume, the nickel matte dispersion is drawn into the slag layer and, as the slag thickness increases, similarly tends to diffuse toward the settling zone of the bath.
(2) The diffusion behavior of nickel matte in the slag layer is significantly influenced by bath circulation. At a slag thickness of 2.0 m, the melt circulation is primarily driven by backflow from the settling zone. In contrast, under the 2.5 m slag thickness condition, the vertical kinetic energy of splashed melt falling back into the reaction zone constitutes the main driving force for the slag circulation. As the slag layer thickness increases, the tendency of matte to migrate toward the settling zone rises, and its degree of dispersion within the slag layer also increases.
(3) The bath circulation induced by splashing reflux is hindered upon reaching the matte layer due to its higher density and thus does not cause significant disturbance to the matte layer in the bottom of the vessel, thus assisting the slag–matte separation.
(4) As the slag thickness increases, the near-wall circulation zone of the melt shifts upward, and the entry point of the ascending air plume into this circulation zone also rises accordingly. This results in a decrease in gas holdup near the tuyere, causing less sulfur removal due to less contact between oxygen and matte sulfur.

## Figures and Tables

**Table 1:** Oprational Parameters of the simulated furnace.

Operational Parameters	Values
Matte layer thickness	1.1 m
Slag layer thickness	1.5 m, 2.0 m, 2.5 m
Tuyere diameter	38 mm
Gas volumetric velocity (single tuyere)	665 m^3^/h
Gas density	1.38 kg/m^3^
Slag density/viscosity	3800 kg/m^3^, 0.10 Pa·s
Nickel matte density/viscosity	4200 kg/m^3^, 0.04 Pa·s
Slag surface tension [31]	0.340 N/m
Matte surface tension [31]	0.360 N/m
Slag–matte interface tension [31]	0.026 N/m

## Data Availability

The original contributions presented in this study are included in the article. Further inquiries can be directed to the corresponding author.

## Nomenclature

α	Volume fraction
ρ	Density
$\vec{v}$	Velocity
p	Pressure
$\vec{g}$	Acceleration of gravity
$\vec{F}$	External body force
subscripts *g*	Gas
subscripts *l*	Liquid
$d_{gl}$	diameter of bubbles or droplets
$C_{D}$	Drag coefficient
$G_{k}$	Turbulence energy due to the mean velocity gradients
$G_{b}$	Turbulence energy due to buoyancy
$\mu_{t}$	Turbulence viscosity
